# Earthquake in the city: using real life gamification model for teaching professional commitment in high school students

**Published:** 2018-09-24

**Authors:** Solmaz Sadat Naghavi Alhosseini, Ata Pourabbasi

**Affiliations:** 1 *Reseacher, Department of Idea Development and Innovation, Endocrinology and Metabolism Research Center, Endocrinology and Metabolism Clinical Sciences Institute, Tehran University of Medical Sciences, Tehran, Iran* *.*; 2 *Assistant Professor, Endocrinology and Metabolism Research Center, Endocrinology and Metabolism Clinical Sciences Institute, Tehran University of Medical Sciences, Tehran, Iran* *.*

**Keywords:** *Professional commitment*, *Gamification*, *Adolescents*, *Education*

## Abstract

Professional commitment plays a significant role in all professions. Moreover, schools are valuable fields for teaching the principles of these concepts especially through novel methods such as gamification. "Earthquake in the city" was implemented in a school in Tehran, Iran, and its effectiveness on learning the concepts of professional commitment was evaluated.

"Earthquake in the city" was built based upon a fantasy scenario occurring in an imaginary city. Each student took on a role in the city (citizen or healthcare provider). After finishing the game, participants were asked about the concept of professional commitment. Their definition was scored by a group of medical ethics experts separately in terms of compliance with the actual definitions and compared with their peers in the control group who did not participate in the game.

A group of 16-year-old teenagers studying in the 11^th^ grade participated in this intervention. The average score of conformity with the actual concept of professional commitment among the case group participants was significantly higher than the average value for the control group (*P* < 0.05).

The results of this study could provide insights to planners and educators engaged in the education system so that gamification can be incorporated as an influential tool to teach the concepts of professional commitment. This experience can also be generalized to other concepts, but designing the appropriate scenario will be the most important component of the intervention in these cases.

## Introduction

In different communities, various occupations are formed in response to the society’s needs and necessitate different skill levels (1). The term professionalism refers to the skills, attitudes, and behaviors that individuals are expected to exhibit in their occupation, including concepts such as maintaining competence, ethical behavior, integrity, honesty, altruism, serving others, following professional codes, justice, and respect for others (2).

Each profession has different moral standards in proportion to the type and sensitivity of the services it provides and its responsibilities toward the society. The understanding of these ethical standards and adherence to them is effective on the moral and professional excellence of individuals (3). One of the most important professions in terms of professional commitment and ethics is the medical profession (4). Healthcare workers must adhere to continuing education throughout their professional life and obtain necessary and cutting edge knowledge and skills; otherwise, the rate of medical errors in the community will be increased (5). There are some controversies regarding the relation between medical errors and professionalism in related studies (6, 7). However, the statistics available in the field of medical errors across countries have resulted in more close consideration of this issue by researchers (5). 

In addition, studies have revealed that increased level of professional commitment among nurses is associated with improved patient-caregiver communication, and better and safer care of the patients. Furthermore, increased professional commitment entails an increase in the quality of patient services (8).

Therefore, professional commitment plays a significant role in the correct implementation of professional and occupational tasks; therefore, teaching of such concepts in different levels of education has been taken into account. Schools do not specifically provide vocational training; however, they are fundamentally inspired by scientific research and most curricula are clearly related to professional occupations. Therefore, it is necessary to use the school platform to teach the principles of professional commitment. Moreover, the use of school environment for teaching bioethical concepts has been previously reported (9). 

One of the new methods that have been taken into consideration in schools is the addition of game elements to instruction (10). Gamification refers to the application of elements of game playing in learning environments, including online collaborative learning environment, and is becoming a common theme among researchers (11). Playing games provides the opportunity to improve students' motivation, to support team work, and to assess them in safe environments (12). Games, in addition to knowledge acquisition, also promote creativity, and logical, mathematical, critical thinking, language, communication and collaboration, and problem-solving skills, and the development of individual and social skills (13).

Professional commitment has an important role in appropriate occupational functioning and its instruction plays an influential role in the professional development of adolescents in the context of schools. Thus, the researchers in this study have investigated the impact of gamification on teaching professional commitment with emphasis on ethical behavior, integrity, honesty, serving others, and respect for others among adolescents using the elements of game to encourage and motivate students to learn effectively. 

To this end, a game called "earthquake in the city" was implemented in one of the schools in Tehran, Iran, and its effectiveness on learning the concepts of professional commitment was evaluated.

## Method

This intervention was carried out in the following stages. Participants of the study were chosen from a group of 16-year-old boys studying in the 11^th^ grade in Tehran. Another group of teenagers at the same educational level of that high school was also participated as the control group. The participants were similar in terms of socio-economic status as well as socio-cultural backgrounds. The approval for this game had already been obtained from the school board.

**Game flow:** *Scenario*

The game "earthquake in the city" was built upon a fantasy scenario occurring in an imaginary city. This game and scenario were validated by an expert panel including some experienced teachers and researchers to ensure that the targeted concepts are considered in the scenario. This scenario was as follows:

*Two days ago, an earthquake with a magnitude of 6.2 occurred in the town. Residential areas have suffered greatly. Since the earthquake occurred at night, the number of victims is high. We are still working to find dead bodies and missing persons. The sources of drinking water have been disturbed; so that the sewage system has encountered a problem and the sewage disposal system has been mixed with groundwater supplies. Most of the people who survived the earthquake are now living in tents. Today, a number of people were infected with various illnesses among which diarrhea, respiratory diseases, and skin disorders are the most prevalent. A 4-year-old child died of gastrointestinal disease yesterday.*

*Rescue operations are not being performed as quickly as they should be and do not have much order. The town has no medical facility to treat the injured. The main hospital of the city has been relatively damaged by the earthquake.*

*Two days after the earthquake, numerous dead bodies are scattered outside the houses, some of them hard to identify with usual methods. Since there is no place to hold dead bodies, they should be inspected and buried quickly because there is a fear that the corpses could transmit other diseases.*

*Children and women who have lost their parents, spouses, and loved ones in this incident are in bad mental conditions and are very inactive; therefore, the town is in danger of an emotional catastrophe.*

*Due to its geological and geographic location and its proximity to a fault, this area of the country is prone to earthquakes; therefore, foreseeing possible aftershocks and earthquakes in other cities is inevitable.*

**Assigned roles**

The roles were extracted according to the game scenario, some of which were related to the in-need inhabitants of the city who were affected by the earthquake while, different roles were designed for service providers. Some service providers were individuals with high professional commitment, and others with low professional commitment. The roles were designed in such a way that for each service provider role there was a corresponding role of an individual needing help. These roles are provided in table 1. 

Each of the roles was written on a piece of paper and placed in a packet on which a number was written.

**Table 1:** Defined roles according to the game scenario

Roles of individuals needing help	Corresponding role of service providers
I am the father of three children and we live together with the family in a tent. The kids are getting sick.	I am a physician working in the internal ward of this hospital. Fortunately, during the earthquake, I was on leave. My leave of absence is approved by the hospital head until the weekend.
I am father of the child who died of diarrhea.	I am a laboratory scientist in charge of the technical diagnostic in the cities’ reference laboratory. It seems that cholera is the common disease associated with diarrhea in this city, and its definitive diagnosis depends upon a laboratory test. But, before setting up my lab, I talked to the head of the city's health network and explained that I would not lift a finger for emergency work.
My kids are thirsty and have no water to drink. Mineral water packages are not distributed among us, so we have to give the kids water from the pits.	I am an environmental health expert working in the health center of this city. Due to the disruption of the sewage treatment, we have been working with a team of volunteers for the last two days to repair the facilities. I hope this can somewhat improve the situation of my fellow citizens.
I live in a tent with my three children. Due to rain, our tent were soaked and last night we had to leave the tent and sleep in the car.	I am a disaster management specialist. The main area of my study is crisis management and the design of health facilities in a state of crisis. However, my area of residency is at a distance of about 700 kilometers from this city.
My mom has been missing since this earthquake. Her body may be among the bodies that cannot be identified.	I am a geneticist and have my PhD in genealogical identification from Johns Hopkins University. Next week, I must speak at a world congress in Turin, Italy. But, now my compatriots are more important.
I have got 2 kids who have lost their mother in this earthquake. They are very restless. I really do not know what to do with them.	I am a psychologist of disaster health. But now I prefer to visit patients in my private counseling clinic that I recently opened in another city and I have to handle paying the rent.
I am the mayor of the neighboring town. I really need to know how likely it is for an earthquake to occur in our city, what facilities we need, and how we should build a field hospital.	I am a geologist with an earthquake orientation. This morning, two of my students and I will departure with a personal car to the city for geological studies and to foresee aftershocks. I hope my science can be of some help to the people of this city.
My brother is trapped under a collapsed wall with a lot of rubble on him. If they do not get him out of the rubble correctly, he may end up with a spinal cord injury forever.	I am a rescue specialist and I am a member of the Rescuers Without Borders Organization. I just returned from a mission in Ethiopia and quickly made my way to this town. I did not even find the chance to get home and visit my family.
I am the governor of the city. We are in a crisis situation. The city hospital is almost destroyed. I do not know if I should use the resources to rebuild the ICU ward quickly, or to provide antibiotics and distribute them among field hospitals.	I am an epidemiologist. My job is to determine the trend of disease outbreaks and identify priority areas. Also, evaluating the effectiveness of interventions is among my specialties. But, now I'm studying to take an overseas scholarship test and do not have time.

**Game process**

Initially, the teacher explained the scenario and the game flow to the students. After that, the envelopes were distributed randomly among the students. Among the participants, only the teacher was aware of the corresponding association between roles according to the numbers written on the envelopes.

After reading the scenario, the teacher announced a random number attributable to a specific role. The student who had that envelope opened it and read his role aloud. Then, the teacher, announced the corresponding number from the lists of roles attributable to service providers, and the student who had that envelope opened the envelope and started reading his role aloud. 

At this stage, the in-need student, with the help of other children, his corresponding was evaluated and the decision was made whether he should be encouraged or punished. If he needed to be encouraged, all pupils would cheer for him, and if punishment was deserved, the in-need role would designed a funny class punishment based on the advice received from other students. The features of this punishment were already announced to students; it was agreed to use a punishment which was not seriously harmful, dangerous, or painful and not to use sharp objects. 

Accordingly, the permissible punishment included sweeping the class, clearing the blackboard without using hands, and standing with both hands and one leg up.

**Assessment**

After finishing the game, the students were asked to take out a piece of paper and write about the concept of professional commitment, and then, hand their notes in. Moreover, they were asked to rate their satisfaction with this class from 0-10 in comparison with the best class they have ever experienced.

The control group was also asked to write statements on the definition of professional commitment.

**Scoring definitions**

A collection of definitions of professional commitment, provided by adolescents in both case and control groups, was compiled as a file. Then, after eliminating the students' names, 2-digit codes were created to help label the pupils. Records were sent to 4 experts of professional ethics, and they were asked to rate the degree of adaptation of the definitions with the actual concept of professional commitment based on a 0-10 scale. The rater was unaware of the order of definitions and their belonging to either the case or control groups. Furthermore, each expert was blinded to the scores from other experts. Finally, the average scores of experts to each definition were considered as the degree of conformity with the actual concept of professional commitment.

***Results***

The participants in this game were a group of 16-year-old boys studying in the 11^th^ grade of high school in Tehran. The game, with the teachers’ explanation at the beginning, lasted 50 minutes. In addition, 15 pupils from the same educational stage were entered into the study as the control group. The participants demographic data are presented in table 2.

**Table 2:** Socioeconomic data of the students

Number of students	26
Mean age of students	16.3 ± 0.2 (mean ± SD)
Time spent in school (hours per day)	8.5
Time spent in school (hours per week)	51

SD= standard deviation

A total of 26 definitions of professional commitment were collected from the participants. These statements and the degree of conformity between the definitions and the actual concept of professional commitment are presented in table 3.

**Table 3:** Statements of pupils on professional commitment and the degree of conformity with the actual concept

Study group	Definition of professional commitment by participants	Degree of conformity with the actual concept of professional commitment
Case (game participants)	Professional commitment means someone, for example a physician, has knowledge and should be faithful to it.	5.50
	That is, everyone in every occupation and profession has obligations that they must adhere to and be responsible for.	6.00
	Imam Ali says: You should desire for others what you desire for yourself. In my opinion, if those professionals put themselves in other people’s shoes (those in need of urgent services), the situation would be completely clear and 90% of them would help.	4.25
	Professional commitment means that I should attend if my presence could be of any help even though it may entail harms for me.	4.75
	The suitable performance of a job that one is responsible for or has the knowledge of, and adherence to the moral and social principles of society	5.75
	Professional commitment is being conscientious and performing one’s duties in any situation (in some cases with self-sacrifice).	5.75
	Complete loyalty to one's occupation at professional levels, such as medicine which the physician is responsible for and should not leave. Such a commitment is clearly more pronounced in clinical disciplines.	6.00
	Completing the assigned tasks as well as doing humanitarian tasks even though they are beyond the scope of duty.	4.25
	Professional commitment means helping the affected people in critical situations.	4.00
	To be called a doctor is a title that cannot be easily achieved. But, when someone reaches this level, he must devote himself entirely to this profession. We swear to serve people as much as we can and do not hesitate in performing our duties. Otherwise, it does not make sense.	6.00
	That is, I do anything I can, even if I have something else to do, otherwise I feel dissatisfied with myself until the end of my life and never forgive myself. A scholar without action is like a bee without honey!	3.25
Study group	**Definition of professional commitment by participants**	**Degree of conformity with** **the actual concept of** **professional commitment**
Control group	In my opinion, professional commitment means to fulfill all our obligations in line with our profession.	4.25
	Adherence to the professional promise	4.25
	Commitment to the principles and rules that are given to us at the beginning of starting a profession and to complete assignments successfully	6.75
	Professional commitment means when we accept something, we should accept it fully and should be responsible for its losses and profits.	5.25
	Accepting a responsibility, and then, fulfillment of the tasks or duties that we have to do	5.50
	I have no idea	.50
	No definition	.50
	It means taking responsibility.	3.50
	It is a promise according to your profession.	3.25
	An important commitment that is fulfilled professionally.	3.00
	Not to sell unreal. As long as my work and duty are not over, I will not leave my job.	3.50
	A professional commitment is an oath and a promise made by individuals, such as doctors, parliamentarians, and presidents, to do their jobs properly.	4.25
	It is adhering to what we are responsible for in a profession, such as a lawyer's commitment to honesty and justice.	4.75
	When we accept a responsibility, we must take it and not to decline our oath.	4.25
	It is the commitment to do right in response to others' trust to our commitment.	4.75

Among the definitions, 4 occupations, including physicians, lawyers, parliamentarians, and the president were mentioned; of which the occupation of a physician was the most frequently referred to (in 4 definitions). Of these definitions, 3 were mentioned by the participating teenagers in the game.

The average score of conformity with the actual concept of professional commitment was 5.04 among the case group participants, which was significantly higher than the average value (3.88) for the control group (*P* < 0.05). The results of this assessment are summarized in table 4.

**Table 4:** Comparison of mean scores of case and control groups in defining the concept of professional commitment

group	N	Mean	Standard deviation	Standard error mean
Case	11	5.0455	0.97991	0.29545
Control	15	3.8833	1.67136	0.43154

In addition, the average satisfaction rate of the participants regarding playing the game in the class was estimated as 8.9 out of 10.

## Discussion

The results of this study showed that gamification could be an effective way to create the concept of professional commitment in adolescents. These results are largely consistent with those reported by other studies targeted at raising the knowledge of adolescents in various fields (14, 15). However, no study has yet incorporated gamification as a way to raise professional commitment. 

Professional commitment is an essential component of adolescent education and schools could provide a suitable and necessary environment for training and institutionalization of the concepts of professional commitment for future professionals.

In order to motivate adolescents to adopt professional behaviors, the educational environment must support and reward professional behaviors and introduce non-professional behaviors using negative roles (16). However, since educational environments may not provide sufficient facilities for instructing professional or non-professional behaviors for various professions, creation of dummy environments through gamification could be a solution to this problem. 

A body of research on the elements of educational games has revealed that gamification increases participation of students in traditional and online learning settings (11); hence, it has many merits. First, it has an intrinsic value because students love games and they are willing to participate in. Second, most games represent a complex situation in which the elements of real life are illustrated in a simple and understandable way which results in sustainable learning and greater participation. Games also provide artificial environments for teenagers in which they can imagine themselves and experience differently (17).

One of the main advantages of gamification is its low cost and the possibility of learning more. In traditional education methods, lecture-based classes are tedious for students. Therefore, the great advantage of gamification technology is its ability to solve this problem (18).

Kapp et al. have widely investigated the impact of gamification on learning, and have shown that the correct implementation of gamification could have an influential impact on student learning outcomes (19). Gamification can greatly promote the learning experience and create a familiar environment for students to develop their abilities (20). A further point of interest is that in the present study gamification was proved to be able to realize educational objectives (i.e., familiarity with the concept of professional commitment). 

It could be claimed that the most important part of the gamification process is designing of a game scenario well-matched with the learning objectives and target audiences. More precisely, the game scenario should be developed and implemented in accordance with the facilities and access level of target audiences which calls for providing facilities and infrastructures.

 For example, many of the game scenarios that can be implemented in cyberspace may not be applicable to a large number of audiences due to limited access. However, more studies are needed to determine the role of a game's scenario in its success in achieving educational objectives.

One of the most important components of using gamification as an educational intervention is how to create motivation in participants for engagement. In the present study we created motivation by defining a system of reward and punishment, which was partly an entertaining activity. Burguillo showed that implementation of the gamification system can increase the amount of task fulfillment in student probably because the competitive environment of games acts as a motivator (21).

According to the authors' experiences, it seems that gamification incorporates thinking and dynamism of the game to increase motivation of users and stimulate their active participation, so improves learning. Games also provide self-assessment tools, such as a scoring mechanism and moving to higher stages (12), which creates incentives for audiences and eliminates application of the other motivation tools (e.g., rewarding). 

Considering the importance of raising students' knowledge about the concepts of professional commitment and the need to use modern educational methods for this purpose, the results of this study could provide insights to planners and educators engaged in the education system; so that gamification can be incorporated as an influential tool to teach the concepts of professional commitment. This experience can also be generalized to other concepts, but the design of the appropriate scenario will be the most important component of the intervention in these cases.

Despite the significant results obtained from this intervention, this study had some limitations including the time limit which led to omitting the educator-student discussion that could assist in establishing the concept of professional commitment during the game. Furthermore, implementation of this game with a larger number of teenagers can provide a better assessment of effectiveness of the intervention.
